# The Life of *Saccharomyces* and Non-*Saccharomyces* Yeasts in Drinking Wine

**DOI:** 10.3390/microorganisms11051178

**Published:** 2023-04-30

**Authors:** Sergi Maicas, José Juan Mateo

**Affiliations:** Departament de Microbiologia i Ecologia, Facultat de Ciències Biològiques, Universitat de València, 46100 Burjassot, Spain

**Keywords:** *Saccharomyces*, non-*Saccharomyces* yeasts, wine, fermentations, enzymes, fruit juices

## Abstract

Drinking wine is a processed beverage that offers high nutritional and health benefits. It is produced from grape must, which undergoes fermentation by yeasts (and sometimes lactic acid bacteria) to create a product that is highly appreciated by consumers worldwide. However, if only one type of yeast, specifically *Saccharomyces cerevisiae*, was used in the fermentation process, the resulting wine would lack aroma and flavor and may be rejected by consumers. To produce wine with a desirable taste and aroma, non-*Saccharomyces* yeasts are necessary. These yeasts contribute volatile aromatic compounds that significantly impact the wine’s final taste. They promote the release of primary aromatic compounds through a sequential hydrolysis mechanism involving several glycosidases unique to these yeasts. This review will discuss the unique characteristics of these yeasts (*Schizosaccharomyces pombe*, *Pichia kluyveri*, *Torulaspora delbrueckii*, *Wickerhamomyces anomalus*, *Metschnikowia pulcherrima*, *Hanseniaspora vineae*, *Lachancea thermotolerans*, *Candida stellata*, and others) and their impact on wine fermentations and co-fermentations. Their existence and the metabolites they produce enhance the complexity of wine flavor, resulting in a more enjoyable drinking experience.

## 1. Introduction

**Figure 1:**
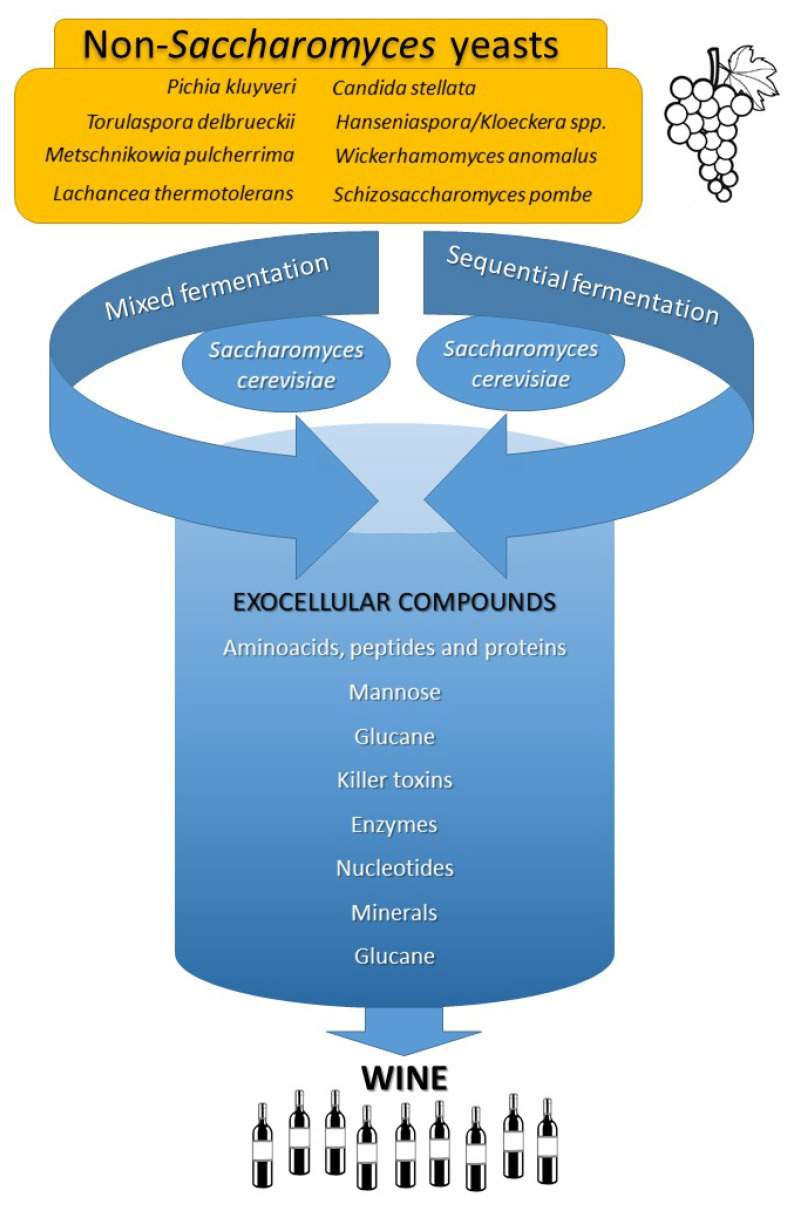
Diagram of wine fermentation by yeasts.

The transformation of fruits into drinking wine through alcoholic fermentation is indeed a fascinating process that has been studied by scientists for many years. This transformation occurs due to the action of yeasts, which convert the sugars present in the fruit juice into alcohol and carbon dioxide [1]. Yeasts are unicellular microorganisms that are found in nature and play an essential role in the fermentation of many foods, including bread, beer, and wine [2]. During the fermentation process, yeasts consume the sugars present in the fruit juice and produce alcohol as a byproduct. The most common yeast species used in wine fermentation is *Saccharomyces cerevisiae*, which is capable of fermenting both glucose and fructose, the two main sugars present in grapes [2]. The choice of the yeast strain used during fermentation can have a significant impact on the final wine product’s aroma, flavor, and overall quality [1,3]. In addition to yeasts, lactic acid bacteria (LAB) also play a secondary role in the wine fermentation process; they are responsible for the malolactic fermentation, a process that occurs after alcoholic fermentation and results in the conversion of malic acid into lactic acid [4,5]. This process can impact the acidity and overall taste of the wine [6]. Thanks to the pioneering work of microbiologists such as Antonie van Leeuwenhoek and Louis Pasteur, the scientific community has a better understanding of the wine fermentation process [7]. With the advancements in scientific knowledge, winemakers and oenologists can now use this knowledge to produce high-quality wines by selecting appropriate yeast strains, controlling the fermentation temperature, and monitoring the fermentation process [2]. Throughout the winemaking process, critical decisions need to be made that impact the fermentation process. Perhaps the first and most challenging decision is whether to add selected yeasts or to rely on the indigenous microbiota to drive the process. While most experts favor adding selected yeasts, allowing the indigenous yeasts to modulate the final wine creates its unique characteristics, setting it apart from other wines [8,9,10,11,12]. Winemakers must be aware of the yeasts present at each stage of production, including at harvest and during the start, mid, and end stages of fermentation [13,14,15,16,17]. It is now universally recognized that to produce quality wine, the joint participation of *S. cerevisiae* and various other yeasts is required, either by their natural participation in vivo or through the addition of commercial preparations containing their lysates [3,18] (Figure 1).

In this review, we will discuss the various yeasts involved in wine fermentation and how they interact to produce a high-quality end product that is appreciated by consumers.

## 2. *Saccharomyces cerevisiae*

In addition to *Saccharomyces cerevisiae*, there are many other yeast species that can be found in grape must and can have an impact on wine quality. Non-*Saccharomyces* yeasts, such as *Hanseniaspora* and *Metschnikowia*, are known to produce a range of aroma and flavor compounds, including esters and higher alcohols, that can contribute to the complexity of wine [19,20,21,22]. However, they are generally less tolerant to high levels of alcohol and can be outcompeted by *S. cerevisiae* during the fermentation process, leading to incomplete or stuck fermentations.

## 3. Non-*Saccharomyces* Yeasts

Recent research has focused on the potential of using mixed cultures of *Saccharomyces* and non-*Saccharomyces* yeasts to improve wine quality. By combining the desirable characteristics of different yeast species, it may be possible to produce wines with more complex and unique flavor profiles. However, the use of mixed cultures can also introduce additional complexity to the fermentation process, as the interactions between different yeast species can be difficult to predict [23,24]. Overall, the choice of yeast strains used in winemaking can have a significant impact on the chemical and sensory properties of the final product. Winemakers must carefully consider the desired flavor profile and fermentation characteristics when selecting yeast strains, and may choose to use mixed cultures to achieve specific goals. Ongoing research in this area may lead to new insights and techniques for optimizing wine quality through yeast selection and fermentation management [1,25,26,27,28]. Furthermore, recent research have shown that non-*Saccharomyces* yeasts can also contribute to the sensory characteristics of wines. For example, some non-*Saccharomyces* yeasts can produce volatile compounds that contribute to fruity, floral or spicy aromas in wine [29,30,31]. Others can produce enzymes that release aroma precursors, which can lead to the development of complex and desirable aromas during aging. Non-*Saccharomyces* yeasts can also contribute to the mouthfeel of wine by producing polysaccharides and glycerol, which can increase the viscosity and perceived body of the wine [32].

In addition to wine production, non-*Saccharomyces* yeasts are used in the production of other fermented foods and beverages, such as beer, cider, mead, and kefir. In beer production, non-*Saccharomyces* yeasts can contribute to the flavor, aroma, and mouthfeel of the final product [33,34,35]. They can also improve fermentation efficiency and reduce the risk of contamination by spoilage microorganisms. Similarly, in cider and mead production, non-*Saccharomyces* yeasts can contribute to flavor and aroma complexity and improve fermentation efficiency. In kefir production, non-*Saccharomyces* yeasts can contribute to the texture and flavor of the final product [23,36].

Overall, the use of non-*Saccharomyces* yeasts in fermentation processes can have a significant impact on the sensory, chemical, and microbial properties of the final product (Table 1). With the help of molecular techniques, researchers can better understand the diversity and functionality of non-*Saccharomyces* yeasts and their potential applications in various fermentation processes. It is worth noting that the use of non-*Saccharomyces* yeasts in winemaking requires careful consideration and control. The use of these yeasts can result in the production of off-flavors, such as hydrogen sulfide, which can affect wine quality. Additionally, these yeasts have varying fermentation kinetics, which can impact the duration and success of the fermentation process. As such, winemakers must carefully select the appropriate non-*Saccharomyces* yeast strains for their specific winemaking goals and monitor the fermentation process closely to ensure optimal results [37,38,39].

Overall, the use of non-*Saccharomyces* yeasts in winemaking is a promising area of research that has the potential to enhance wine quality and reduce the use of synthetic additives. However, further studies are needed to fully understand the mechanisms behind the interactions between different yeast species and their impact on wine quality. In addition to their potential to improve the sensory quality of fermented beverages, non-*Saccharomyces* yeasts have also been shown to have potential health benefits. For example, some non-*Saccharomyces* yeasts have been found to produce high levels of β-glucans, which have immunomodulatory properties and have been shown to improve gut health [81]. Other non-*Saccharomyces* yeasts have been reported to produce anti-inflammatory and anti-cancer compounds, such as exopolysaccharides and peptides [82,83]. Therefore, there is increasing interest in the use of non-*Saccharomyces* yeasts in the development of functional foods and nutraceuticals. Furthermore, the use of non-*Saccharomyces* yeasts in fermentation has environmental benefits, as they can reduce the use of chemical additives and decrease the carbon footprint of the fermentation process [37]. In addition, some non-*Saccharomyces* yeasts have been shown to have a positive impact on soil health, as they are able to solubilize soil nutrients and enhance plant growth [84].

Overall, the use of non-*Saccharomyces* yeasts in fermentation has significant potential for improving the sensory quality, health benefits, and environmental sustainability of fermented beverages and other products. Further research is needed to fully understand their fermentative capabilities and the mechanisms underlying their health-promoting properties [58,85,86].

### 3.1. *Schizosaccharomyces pombe*

Initially considered a spoilage yeast, *Schizosaccharomyces* has been successfully used industrially in the fermentation of sugar cane during rum making and palm and cocoa fermentation, and there are high hopes for its use in the wine industry [40]. Malolactic fermentation is often used to reduce the malic acid content in musts and wines, especially in the production of red wines, although it is sometimes a very complicated process due to the growth requirements of the bacteria used [40]. The use of *Sc. pombe* could become an invaluable new tool for grapes from wine-growing regions where malic acid is present in excessive concentrations, although few commercial strains are available due to their high rate of acetic acid production (about 1 g/L). Mixed and sequential cultures with *Saccharomyces* have been used to reduce the negative effects of *Schizosaccharomyces* spp. strains [87]. However, *Sc. pombe* has much greater potential than just its ability to reduce malic acid content and ferment sugar. Some researchers are using *Sc. pombe* to decrease gluconic acid [41]. Another application is aging on lees due to the greater autolytic release of polysaccharides than with *Saccharomyces* [42]. The ability of *Sc. pombe* to reduce 4-ethylphenol in wine due to its high adsorption capacity has also been studied [43]. Furthermore, urease activity is also of great interest in relation to food safety. Urea is the main precursor of ethyl carbamate, so reducing urea content could reduce ethyl carbamate, which is one of the main food safety problems in modern oenology [44]. Furthermore, the use of *Schizosaccharomyces* could limit the risk of biogenic amines [87], which are notorious for causing physiological problems in humans [88]. In addition, *Schizosaccharomyces* produces a large amount of pyruvic acid, and the significant hydroxycinnamate decarboxylase activity of *Schizosaccharomyces* favors the formation of vinylphenolic pyranoanthocyanins [87,89].

### 3.2. *Pichia kluyveri*

The various species of *Pichia* are fascinating non-*Saccharomyces* yeasts in oenology and are typically present in must fermentations, directly linked to wine [55]. The most frequently cited species in literature are *P. fermentans* [74,75], *Pichia membranifaciens* [90], *Pichia occidentalis* [91], *Pichia terricola*, *Pichia manshurica*, *Pichia kudriavzevii*, and *Pichia kluyveri*. The frequency of *Pichia* in grapes is lower than that of *S. cerevisiae* (28%) and other species such as *Hanseniaspora uvarum* (44%). The frequency varies from 0.12% for *P. occidentalis* to 4.7% for *Pichia anomala*. Other *Pichia* species commonly isolated from grapes are *P. manshurica* (2.81%), *P. membranifaciens* (0.98%), and *P. kudriavzevii* (0.85%) [92]. As a result, there are no adequate selection methods available, mainly because of these low frequencies that do not contribute to the development of commercial strains. However, *P. kluyveri* is perhaps the most researched species, for which commercial preparations are available. These species are known for their abilities to enhance the composition of aromatic compounds, providing thiols, terpenes, and fruity esters. When added to the fermentation process, they enhance rose petal and floral aromas, contributing to the overall bouquet of the wine, and the varietal and thiol aromas [72,73]. Marketers of wine suggest a sequential use of starter cultures, adding *P. kluyveri* first and after 48 h of fermentation, *S. cerevisiae*, which is better adapted to high ethanol concentrations and will complete the fermentation process. Biochemically, *Pichia* species ferment glucose but not other sugar molecules easily. Overall, the various *Pichia* species are gaining increasing interest in oenology [85,93,94].

### 3.3. *Torulaspora delbrueckii*

*Torulaspora delbrueckii* is one of the most commonly used non-*Saccharomyces* yeasts in oenology. It is recognized for its ability to improve the quality of wine and significantly reduce volatile acidity, particularly in musts with high sugar concentrations [95]. Winemakers consider this yeast to be a good option for optimizing certain wine parameters, as compared to those produced with *S. cerevisiae*. Specifically, *T. delbrueckii* is attributed with a lower acetic acid production capacity, lower ethanol concentration, higher amounts of glycerol, increased release of mannoproteins and polysaccharides, and greater potential for malolactic fermentation. In addition, this yeast has been observed to produce a higher number of desirable aroma compounds such as fruity esters lactones, thiols, and terpenes, while reducing the production of undesirable aroma compounds such as higher alcohols [31,66,96]. From an organoleptic perspective, *T. delbrueckii* contributes significantly to the reduction of esters and, at the same time, increases the concentration of minor esters and lactones, making it an important factor in the production of white wine [95]. In this way, the action of *T. delbrueckii* reduces the intensity of the fresh fruit aromas that are characteristic of young wines. At the same time, it increases the aromas of raisined fruit and sweetness, giving the wine aromatic characteristics associated with wines that have been produced over a longer period of time [19]. For this reason, some wine producers consider that *T. delbrueckii* is not a recommended yeast for making young white and rosé wines, as they are less aromatic and more evolved than those produced with *Saccharomyces*.

The effect attributable to *T. delbrueckii* varies depending on its degree of involvement in fermentation. When *S. cerevisiae* is involved, it is usually weak. To increase its effect, killer strains of *T. delbrueckii* are used, thus achieving a more reproducible effect of these yeasts on the final aroma of the wine [66]. It is known that *T. delbrueckii* has a lower fermentation potential and a lower growth rate than *S. cerevisiae* when used under normal fermentation conditions. When using killer strains, the environmental conditions are modified.

On the other hand, red wine production differs in certain aspects that may affect the growth of *T. delbrueckii* during fermentation and the quality of the wine. Frequent punching (oxygenation of the grape skins) allows for a higher availability of oxygen in the fermentation of red wines in comparison with white wines. This may benefit *T. delbrueckii*, since respiration is more relevant to its metabolism than that of *S. cerevisiae*, so the former grows worse than the latter under strictly anaerobic conditions [97].

However, the alcohol content is usually higher in red wines than in white wines. This negatively affects the ability of *T. delbrueckii* to dominate and complete fermentation, especially in the presence of *S. cerevisiae*, which is a yeast better adapted to high ethanol levels. Nevertheless, the initial amount of wild microorganisms is usually higher in red wine fermentation than in white wine fermentation. Red wine fermentation takes place in the presence of skins, which hold together more bacteria that carry out malolactic fermentation [5]. In addition, the presence of skins in the must provides additional nutrients, which can improve the fermentative capacity of *T. delbrueckii* and make it more competitive with *S. cerevisiae* [98].

### 3.4. *Wickerhamomyces anomalus*

The presence of *W. anomalus* during the fermentation process can have both positive and negative effects on the final wine product. On one hand, research has shown that this yeast contributes to the production of desirable aromas and flavors in wine, such as floral and fruity notes [52]. However, uncontrolled growth of *W. anomalus* can lead to spoilage and off-flavors in the wine [31,99,100]. To prevent the growth of spoilage yeasts such as *W. anomalus*, sulfites (e.g., sulfur dioxide) are commonly added to the wine by winemakers [101]. The addition of sulfites helps inhibit the growth of unwanted microorganisms, preserving the wine’s freshness and flavor. Nevertheless, some individuals are sensitive to sulfites, and high levels of sulfites in wine can trigger allergic reactions [102]. As a result, winemakers are exploring alternative approaches for preventing spoilage, such as using non-sulfite antimicrobial agents or implementing more frequent monitoring and testing during wine production [103].

Enzymes produced by *W. anomalus*, such as glycosidases, can contribute to the release of aromatic compounds in wine by hydrolyzing glycosidic bonds that are present in grape precursors [55]. These glycosidic bonds can mask the aromatic compounds, making them less volatile and therefore less perceptible to the human nose. By releasing these compounds, *W. anomalus* can have a significant impact on the aroma of wine. For example, β-D-glucosidase can hydrolyze glycosides of monoterpene alcohols, which are important contributors to floral and fruity aromas in wine [104]. Strains identified as *W. anomalus* or its former names have been reported to produce glycosidases such as β-D-glucosidase, α-L-arabinofuranosidase, α-L-rhamnosidase, and β-D-xylosidase, which are involved in the release of compounds aromatics from grape precursors [32]. The production of these enzymes by *W. anomalus* can therefore enhance the aroma profile of wine, potentially making it more complex and interesting. As such, strains of *W. anomalus* have been studied for their potential use in the oenological industry as a source of enzymes for improving wine aroma [105].

### 3.5. *Metschnikowia pulcherrima*

*Metschnikowia pulcherrima* is a non-*Saccharomyces* yeast present in various ecological niches, including the surface of grapes. Morphologically, its shape is ovoid to ellipsoidal with a size of 2.5 μm × 4^−10^ μm. The diploid cells of this species propagate vegetatively by budding. Under certain anaerobic conditions it can form pseudohyphae. It can form one to two lance-shaped (acicular/threadlike) spores. Its colonies are cream-colored and produce a reddish-brown soluble pigment called pulcherrimina, characteristic of this species, which gives color to the colonies and diffuses towards the medium. Strains of *M. pulcherrima* can be identified using selective and differential media: they show positive activity of the enzyme β-glucosidase, expression indicated by the use of arbutin as carbon source in agar plates; and proteolytic activity [106]. It grows well in media such as yeast extract peptone dextrose (YPD) or L-lysine. On the other hand, it shows very weak growth on nitrate agar [56]. Regarding the metabolism of *M. pulcherrima*, it is known that it can use glucose, sucrose, fructose, galactose, and maltose as carbon sources. However, it seems that with lactose it shows weak or non-existent growth. On the other hand, it can grow adequately at low temperatures of 15 to 20 ${}^{{}^{\circ}}$C and with a pH between 3 and 6 [56]. *M. pulcherrima* is one of the non-*Saccharomyces* yeast species capable of expressing more extracellular hydrolytic enzymes, highlighting the following: amylase, cellulase, glucanase, β-glucosidase, β-lyase, lipase, lichenase, pectinase, protease, sulfite reductase, and xylanase [58].

*M. pulcherrima* is considered a promising candidate for producing wine with low ethanol content. Previous studies have demonstrated that inoculating this yeast in combination with other yeasts, such as *Saccharomyces uvarum*, can result in wines with lower alcohol concentrations. However, *M. pulcherrima* has a lower fermentation power compared to other non-*Saccharomyces* yeasts [56]. Moreover, the reduction in alcohol production appears to be additive when used in combination with other yeasts, but competition and interactions with the autochthonous microbiota of grapes during fermentation can limit the expected results [60].

### 3.6. *Hanseniaspora/Kloeckera* spp.

Yeasts belonging to the genus *Hanseniaspora* are ascomycetes that are easily identified by their characteristic apiculate shape under the microscope, resulting from bipolar budding. This genus is part of the non-*Saccharomyces* yeasts, which are frequently isolated during the initial stages of fermentation. These yeasts can also be found on the surface of grapes, in soil, and in the winery environment, including harvesting machinery and fruit processing equipment [76,77]. Apiculate yeasts belonging to the genus *Hanseniaspora* are prevalent on grape surfaces. While *H. uvarum* is known for its abundant presence on grapes and its negative impact on wine quality due to high volatile acidity production, less is known about *H. vineae*, which is better adapted to fermentation. Studies have reported that *H. vineae* has enzymatic activity [77,78] and is capable of producing high levels of desirable aromatic compounds [79,107], enhancing the sensory properties of wines produced on an industrial scale. Additionally, proteolytic activity has been observed in *Hanseniaspora* isolates [62].

### 3.7. *Lachancea thermotolerans*

*Lachancea thermotolerans*, which was previously known as *Kluyveromyces thermotolerans*, is a yeast commonly found in various natural environments, including grapes. Its elliptical shape makes it indistinguishable from *S. cerevisiae* under light microscopy [108]. This yeast reproduces sexually with the formation of 1–4 ascospores and has been available as an active dry yeast since 2012, marketed by Christian Hansen (CHR-Hansen) to enhance the sensory characteristics of wines. A recent review [64] highlights its effects on acidity, aromatic profile, and polyalcohol production. *L. thermotolerans* has a moderate fermentation power (4–10% *v*/*v*) and is recommended for mixed or sequential use with other species such as *S. cerevisiae* or *Sc. pombe*, to allow for complete fermentation of the must’s sugars. One of the most noteworthy properties of this yeast is its ability to produce lactic acid, which can effectively enhance the acidity and pH of wines in a stable manner, as this acid remains unchanged during wine aging and stabilization. Additionally, the pH modification capability is crucial, with some strains able to exceed 0.5 pH units under real vinification conditions, particularly when used in sequential fermentations on crushed red grapes, in the presence of solid parts [64]. This is due to the fact that most of the acidification by *L. thermotolerans* occurs during the initial stages of fermentation, making it a strong competitor against other wine yeast species, and also because of its excellent tolerance to ethanol. In addition to its enzymatic activities, *L. thermotolerans* also produces a range of volatile compounds that contribute to the aroma of wines. These include esters, such as isoamyl acetate and ethyl lactate, as well as higher alcohols, such as isoamyl alcohol and 2-phenylethanol [65]. It has also been reported to produce sulfur compounds, such as thiol precursors, which can contribute to the tropical and citrus fruit notes in wine [64]. Overall, *L. thermotolerans* is considered a promising non-*Saccharomyces* yeast for improving the sensory properties of wines, especially in terms of acidity and aroma. Recent studies describe that it can favor the release of terpenes and volatile thiols [109]. Various works also show a positive effect of the use of *L. thermotolerans* on glycerol contents [66]. Glycerol is the second quantitatively most important fermentative metabolite after ethanol and has a certain effect on wine smoothness and structure [110].

Further studies have aimed to explore the biocompatibilities between *L. thermotolerans* and *Hanseniaspora* spp. in co-inoculation, using different types of nutrients and considering the effect on yeast assimilable nitrogen at low (16 °C) and medium temperatures SO_2_ (50 mg/L) for improving the sensory profile [111].The behavior of these yeasts was evaluated, and significant results were obtained on the population count, with higher populations of *Hanseniaspora* spp. with respect to *L. thermotolerans*. Not surprisingly, fermentations with *L. thermotolerans*/*H. vineae*, showed inhibition of acidification, generating up to 0.41 g/L of lactic acid. On the contrary, a synergistic effect was observed when *L. thermotolerans*/*H. opuntiae* was used, achieving 2.44 g/L of lactic acid and a pH reduction of up to 0.16 [111].

### 3.8. *Candida stellata*

Non-*Saccharomyces* yeasts, such as *Candida* spp., are becoming increasingly important in the industry due to their unique fermentative behavior. *Candida* species have been identified as potential candidates for the fermentation of wine and beer [112]. In particular, *C. stellata* is frequently isolated from grape must and can survive throughout spontaneous wine fermentation for extended periods of time [113]. Studies on the fermentative activity of *C. stellata* have shown that it can have a positive impact on the taste and flavor of alcoholic beverages [67]. In addition to its ability to positively affect the taste and flavor of alcoholic beverages, *C. stellata* also exhibits unique metabolic characteristics. It is known to have a strong preference for fructose and high osmotic pressure environments. Under anaerobic conditions or limited oxygen supply, it undergoes alcoholic fermentation, but under completely aerobic conditions, a mixed respiro-fermentative metabolism is observed. This metabolic behavior is influenced by the concentration of oxygen and glucose in the fermentation medium. When conditions are conducive to sugar fermentation, major fermentative compounds such as ethanol, acetic acid, and glycerol are produced, along with small amounts of higher alcohols, esters, volatile fatty acids and carbonyl compounds. Despite these metabolic losses, the complete fermentation of hexose by yeast can still produce 94–96% of the theoretical yield of ethanol [69,70,71,114].

### 3.9. Limitations in the Research Field and Future Perspectives

While the use of non-*Saccharomyces* yeasts in winemaking shows great potential, there are still some limitations in the research field that need to be addressed. One of the biggest limitations is the lack of understanding of how non-*Saccharomyces* yeasts interact with *S. cerevisiae* during co-fermentation. While it is known that non-*Saccharomyces* yeasts can contribute to wine flavor and aroma complexity, more research is needed to determine the specific mechanisms involved in this process [115,116]. Another limitation is the lack of commercial availability of non-*Saccharomyces* yeasts. While some non-*Saccharomyces* yeasts are available commercially, there is still a limited selection compared to the vast diversity of non-*Saccharomyces* yeasts present in nature. This limitation has led to a lack of standardization in the use of non-*Saccharomyces* yeasts in winemaking, making it difficult to compare the effects of different yeasts on wine quality [117,118].

Despite the limitations in the research field, the use of non-*Saccharomyces* yeasts in winemaking shows great potential for the future. With advances in molecular biology and genetic engineering, it may be possible to develop new non-*Saccharomyces* yeasts with specific traits that are desirable for winemaking. For example, it may be possible to develop non-*Saccharomyces* yeasts that are better able to survive in the harsh conditions of winemaking, or that are better able to compete with *S. cerevisiae* during co-fermentation. The use of these new yeasts will be conditioned to legal aspects in some countries [119,120].

Another potential avenue for research is the use of mixed cultures of non-*Saccharomyces* yeasts. While much of the current research focuses on the use of non-*Saccharomyces* yeasts in combination with *S. cerevisiae*, it may be possible to develop mixed preparations [97,121].

## 4. Conclusions

Life in wine is not easy for microorganisms, and the early stages of wine fermentation can be especially challenging. In the first stage, a very sweet juice with high concentrations of sugars provides a placid environment in which yeasts can develop and carry out different metabolic activities, mainly alcoholic fermentation. In such an environment, *S. cerevisiae* could be the dominant yeast, in the absence of high competition. However, this is often not the case. There are numerous yeast genera present, mainly from other genus grouped under the term non-*Saccharomyces*, which can outcompete *S. cerevisiae* in the early stages of fermentation. These non-*Saccharomyces* yeasts develop and produce numerous aromatic compounds that ultimately define the resulting wine. They are responsible for much of the complex aroma and flavor profiles that we associate with fine wines. This process goes hand in hand with an increasing production of alcohol. However, as the alcohol concentration in the wine increases, the environment becomes more hostile to most yeast species. High amounts of alcohol are unbearable for almost all yeasts, except for *S. cerevisiae*, which has developed a remarkable ability to tolerate high levels of alcohol. As a result, *S. cerevisiae* has ended up being the triumphant yeast in this world. It is able to outcompete other yeasts and continue to ferment the wine until most of the sugar has been converted to alcohol. During this stage, the yeasts produce more alcohol, and the wine becomes more acidic. Eventually, the alcohol concentration reaches a point where it becomes toxic even for *S. cerevisiae*, and the fermentation comes to a natural end. The resulting wine is a complex mixture of different aromatic compounds, acids, and other components, all of which contribute to its unique sensory profile.

## Figures and Tables

**Table 1:** Yeast enzymatic activities of non-*Saccharomyces* yeasts in wine.

Yeast	Enzymatic Activity in Wine	References
*Schizosaccharomyces pombe*	Reduce malic acid content	[40]
	Great autolytic release of polysaccharides	[41,42]
	Reduce wine 4-ethylphenol concentration	[43,44]
*Torulaspora delbrueckii*	Reduce volatile acidity	[45,46]
	Increase the concentration of some minor lactones and esters	[47,48]
	Killer strains	[49,50]
*Wickerhamomyces anomalus*	Tolerate up to 12.5% (*v*/*v*) ethanol	[51,52]
	Produce lethal toxins	[53,54]
	Source of different enzymes	[32,55]
*Metschnikowia pulcherrima*	Production of pulcherrimin	[56,57]
	β-glucosidase activity	[58,59]
	Good candidate for to obtaining wine with low ethanol content	[60,61]
*Lachancea thermotolerans*	Produce lactic acid	[62,63]
	Produce low volatile acidity	[64,65]
	Express extracellular enzymatic activities	[65,66]
*Candida stellata*	Positively affect the taste and flavor of alcoholic beverages	[67,68]
	Strong fructophilic and osmophilic character	[69,70]
	Positive Crabtree yeast	[70,71]
*Pichia kluyveri*	Supply of thiols, terpenes and fruity esters	[72,73]
	Ferment glucose but hardly other sugar molecules.	[74,75]
	Produce extracellular enzymes	[40,76]
*Hanseniaspora*	High volatile acidity production	[76,77]
	Produce extracellular enzymes	[78,79]
	Proteolitic activities	[62,80]

## Data Availability

No new data available.

## References

[1] Pretorius I.S. (2000). Tailoring wine yeast for the new millennium: Novel approaches to the ancient art of winemaking. Yeast.

[2] Fleet G.H. (2003). Yeast interactions and wine flavour. Int. J. Food Microbiol..

[3] Maicas S. (2021). Advances in wine fermentation. Fermentation.

[4] Barata A., Malfeito-Ferreira M., Loureiro V. (2012). The microbial ecology of wine grape berries. Int. J. Food Microbiol..

[5] Maicas S. (2001). The use of alternative technologies to develop malolactic fermentation in wine. Appl. Microbiol. Biotechnol..

[6] Lonvaud-Funel A. (1999). Lactic acid bacteria in the quality improvement and depreciation of wine. Antonie van Leeuwenhoek.

[7] Boulton R. (1996). The application of microbiology in the wine industry. Crit. Rev. Biotechnol..

[8] Di Maio S., Polizzotto G. (2021). Native yeasts for wine fermentation: A review. Fermentation.

[9] Belda I., Ruiz J., Alastruey-Izquierdo A., Navascués E., Marquina D., Santos A., Calderón F. (2020). Unraveling the contribution of indigenous and co-inoculated yeast to wine fermentation. Food Microbiol..

[10] Knight S., Klaere S., Fedrizzi B., Goddard M.R., Jiranek V. (2018). Regional microbial signatures positively correlate with differential wine phenotypes: Evidence for a microbial aspect to terroir. Sci. Rep..

[11] Lencioni L., Romani C., Gobbi M., Capece A., Tirelli A. (2020). *Candida zemplinina* and *Saccharomyces cerevisiae* mixed fermentations to enhance complexity and variability of wines from Malvasia grapes. Food Microbiol..

[12] Longo R., Capece A., Comi G. (2018). Selection of indigenous *Saccharomyces cerevisiae* strains to make Amarone wine. Ann. Microbiol..

[13] Rojo Bezares B., Sanchez-Patàn F., Palop M.L., López-Alfaro I. (2018). Diversity and dynamics of yeast communities in wine fermentation processes. Ann. Microbiol..

[14] Santamaría P., López-Rituerto E., Garijo P. (2019). Wine fermentation microbiome: A landscape from different Spanish wine denominations. Food Microbiol..

[15] Deed R.C., Fedrizzi B., Gardner J.M., Jiranek V., Borneman A.R. (2021). Variability of wine yeast strain populations across seasons and locations in an Australian vineyard. Front. Microbiol..

[16] Fuss A., du Toit M. (2021). The impact of grape variety, harvest year and yeast strain on the yeast population during the fermentation of South African Pinotage wines. S. Afr. J. Enol. Vitic..

[17] Maicas S. (2020). The role of yeasts in fermentation processes. Microorganisms.

[18] Tofalo R., Perpetuini G., Rossetti A.P., Gaggiotti S., Piva A., Olivastri L., Cichelli A., Compagnone D., Arfelli G. (2022). Impact of *Saccharomyces cerevisiae* and non-*Saccharomyces* yeasts to improve traditional sparkling wines production. Food Microbiol..

[19] Ciani M., Capece A., Comitini F., Canonico L., Siesto G., Romano P. (2016). Yeast interactions in inoculated wine fermentation. Front. Microbiol..

[20] Tofalo R., Schirone M., Fasoli G., Perpetuini G., Perpetuini F., Corsetti A., Suzzi G. (2019). The contribution of non-*Saccharomyces* yeasts to wine aroma. Ann. Microbiol..

[21] Medina-Trujillo L., González-Robles I.A., Durán-Cruz M.J., Escalante-Minakata P. (2018). Diversity and dynamics of yeast communities during fermentation of “Criolla” grape musts. Front. Microbiol..

[22] Masneuf-Pomarède I., Bely M., Marullo P., Albertin W. (2019). The genetics of non-conventional wine yeasts: Current knowledge and future challenges. Front. Microbiol..

[23] Jolly N.P., Varela C., Pretorius I.S. (2014). Not your ordinary yeast: Non-*Saccharomyces* yeasts in wine production uncovered. FEMS Yeast Res..

[24] Pérez-Martín F., Medina-Trujillo L., Escott C., Rodríguez M.E., García-Martínez T. (2019). Use of non-*Saccharomyces* yeasts alone or in combination with *Saccharomyces cerevisiae* during alcoholic fermentation of Tempranillo wine: Impact on aroma compounds. Food Res. Int..

[25] González-Arenzana L., Moreno-Arribas M.V. (2018). Wine microbiome: A dynamic world of microbial interactions. Crit. Rev. Food Sci. Nutr..

[26] Jolly N.P., Augustyn O.P.H. (2018). The use of non-*Saccharomyces* yeast species in wine production. S. Afr. J. Enol. Vitic..

[27] Padilla B., Gil J.V., Manzanares P. (2018). Challenges of the Non-Conventional Yeast *Wickerhamomyces anomalus* in Winemaking. Fermentation.

[28] Varela C., Borneman A.R. (2018). Yeast found on grapes and in musts during winemaking. Yeast.

[29] Grangeteau C., Gerhards D., Rousseaux S., von Wallbrunn C., Alexandre H., Guilloux-Benatier M. (2020). Contribution of non-*Saccharomyces* yeasts to wine volatile and sensory diversity: A study on *Lachancea thermotolerans*, *Metschnikowia pulcherrima* and *Candida zemplinina*. Food Microbiol..

[30] Liu J., Zhu B., Zhang Z. (2021). Impact of Non-*Saccharomyces* Yeasts on Volatile Compound Profiles in Wine Fermentation: A Review. Front. Bioeng. Biotechnol..

[31] Varela C. (2019). Sensory impact of non-*Saccharomyces* yeasts in wine. Curr. Opin. Food Sci..

[32] Padilla B., Gil J.V., Manzanares P. (2016). Past and Future of Non-*Saccharomyces* Yeasts: From Spoilage Microorganisms to Biotechnological Tools for Improving Wine Aroma Complexity. Front. Microbiol..

[33] Vegi A., Alexopoulos A., Chrysanthopoulos P.K., Koutinas A.A., Kookos I.K. (2018). The Potential of Non-*Saccharomyces* Yeasts in Beer Production. Fermentation.

[34] Fleet G.H. (2019). Non-*Saccharomyces* yeasts: The underestimated and indispensable contribution of early co-fermentations in wine. J. Ind. Microbiol. Biotechnol..

[35] Contreras A., Escott C., Cuesta I., Torija M.J., Gonzalez-Arenzana L., Garijo P., Lopez-Alfaro I., Lopez R., Suarez-Lepe J.A. (2021). Exploring the potential of non-*Saccharomyces* yeasts for cider fermentation: A comparative study with *Saccharomyces cerevisiae*. Food Microbiol..

[36] Gallone B., Steensels J., Prahl T., Soriaga L., Saels V., Herrera-Malaver B., Merlevede A., Roncoroni M., Voordeckers K., Miraglia L. (2016). Domestication and divergence of *Saccharomyces cerevisiae* beer yeasts. Cell.

[37] Liu S.Q. (2018). Non-*Saccharomyces* Yeasts: The Secondary Players inWine Fermentation. Wine Science: Principles and Applications.

[38] Schüller D., Lombardero J., Guillamón J.M., del Olmo M.L. (2019). Exploring the potential of non-*Saccharomyces* yeasts for the reduction of alcohol content in wine. Appl. Microbiol. Biotechnol..

[39] Padilla B., Gil J.V., Manzanares P. (2020). The impact of non-*Saccharomyces* yeast species in the production of sparkling wines. Foods.

[40] Fleet G.H. (2008). Wine yeasts for the future. FEMS Yeast Res..

[41] Peinado R.A., Moreno J.J., Maestre O., Mauricio J.C. (2007). Removing gluconic acid by using different treatments with a *Schizosaccharomyces pombe* mutant: Effect on fermentation byproducts. Food Chem..

[42] Palomero F., Morata A., Benito S., Calderón F., Suárez-Lepe J.A. (2009). New genera of yeasts for over-lees aging of red wine. Food Chem..

[43] Palomero F., Ntanos K., Morata A., Benito S., Suárez-Lepe J. (2011). Reduction of wine 4-ethylphenol concentration using lyophilised yeast as a bioadsorbent: Influence on anthocyanin content and chromatic variables. Eur. Food Res. Technol..

[44] Benito S., Hofmann T., Laier M., Lochbühler B., Schüttler A., Ebert K., Fritsch S., Röcker J., Rauhut D. (2015). Effect on quality and composition of Riesling wines fermented by sequential inoculation with non-*Saccharomyces* and *Saccharomyces cerevisiae*. Eur. Food Res. Technol..

[45] Sgouros G., Mallouchos A., Dourou D., Banilas G., Chalvantzi I., Kourkoutas Y., Nisiotou A. (2023). *Torulaspora delbrueckii* May Help Manage Total and Volatile Acidity of Santorini-Assyrtiko Wine in View of Global Warming. Foods.

[46] Silva-Sousa F., Fernandes T., Pereira F., Rodrigues D., Rito T., Camarasa C., Franco-Duarte R., Sousa M.J. (2022). *Torulaspora delbrueckii* Phenotypic and Metabolic Profiling towards Its Biotechnological Exploitation. J. Fungi.

[47] Wang R., Sun J., Lassabliere B., Yu B., Liu S.Q. (2020). Fermentation characteristics of four non-*Saccharomyces* yeasts in green tea slurry. Food Microbiol..

[48] Ramírez M., Velázquez R., Maqueda M., Zamora E., López-Piñeiro A., Hernández L.M. (2016). Influence of the dominance of must fermentation by *Torulaspora delbrueckii* on the malolactic fermentation and organoleptic quality of red table wine. Int. J. Food Microbiol..

[49] Corbu V.M., Csutak O. (2022). Molecular and Physiological Diversity of Indigenous Yeasts Isolated from Spontaneously Fermented Wine Wort from Ilfov County, Romania. Microorganisms.

[50] Ramírez M., Velázquez R., López-Piñeiro A., Martínez A. (2021). Genome Features of a New Double-Stranded RNA Helper Virus (LBCbarr) from Wine *Torulaspora delbrueckii* Killer Strains. Int. J. Mol. Sci..

[51] Heard G.M., Fleet G.H. (1985). Growth of Natural Yeast Flora during the Fermentation of Inoculated Wines. Appl. Environ. Microbiol..

[52] Díaz C., Molina A.M., Nähring J., Fischer R. (2013). Characterization and Dynamic Behavior of Wild Yeast during Spontaneous Wine Fermentation in Steel Tanks and Amphorae. Biomed. Res. Int..

[53] Sabel A., Martens S., Petri A., König H., Claus H. (2014). *Wickerhamomyces anomalus* AS1: A new strain with potential to improve wine aroma. Ann. Microbiol..

[54] Nascimento B.L., Martelli E.C., da Silva J.C., Delabeneta M.F., Rosseto L.R., Junges D.S., Paris A.P., Persel C., Paula C.R., Simão R.C. (2022). Inhibition of *Klebsiella pneumoniae* carbapenemases by mycocins produced by *Wickerhamomyces anomalus*. Arch. Microbiol..

[55] Madrigal T., Maicas S., Mateo Tolosa J.J. (2013). Glucose and Ethanol Tolerant Enzymes Produced by *Pichia* (*Wickerhamomyces*) Isolates from Enological Ecosystems. Am. J. Enol. Vitic..

[56] Morata A., Loira I., Escott C., del Fresno J.M., Bañuelos M.A., Suárez-Lepe J.A. (2019). Applications of *Metschnikowia pulcherrima* in Wine Biotechnology. Fermentation.

[57] Mažeika K., Šiliauskas L., Skridlaitė G., Matelis A., Garjonytė R., Paškevičius A., Melvydas V. (2021). Features of iron accumulation at high concentration in pulcherrimin-producing *Metschnikowia* yeast biomass. J. Biol. Inorg. Chem..

[58] Barbosa C., Lage P., Esteves M., Chambel L., Mendes-Faia A., Mendes-Ferreira A. (2018). Molecular and Phenotypic Characterization of *Metschnikowia pulcherrima* Strains from Douro Wine Region. Fermentation.

[59] Qin T., Liao J., Zheng Y., Zhang W., Zhang X. (2021). Oenological Characteristics of Four Non-*Saccharomyces* Yeast Strains With/*beta*-Glycosidase Activity. Front. Microbiol..

[60] Puškaš V.S., Miljić U.D., Djuran J.J., Vučurović V.M. (2020). The aptitude of commercial yeast strains for lowering the ethanol content of wine. Food Sci. Nutr..

[61] Postigo V., Sanz P., Garc’ia M., Arroyo T. (2022). Impact of Non-*Saccharomyces* Wine Yeast Strains on Improving Healthy Characteristics and the Sensory Profile of Beer in Sequential Fermentation. Foods.

[62] Mateo J.J., Maicas S., Thiessen C. (2015). Biotechnological characterisation of exocellular proteases produced by enological *Hanseniaspora* isolates. Int. J. Food Sci. Technol..

[63] Vicente J., Baran Y., Navascu’es E., Santos A., Calder’on F., Marquina D., Rauhut D., Benito S. (2022). Biological management of acidity in wine industry: A review. Int. J. Food Microbiol..

[64] Morata A., Loira I., Tesfaye W., Bañuelos M.A., González C., Suárez Lepe J.A. (2018). *Lachancea thermotolerans* Applications in Wine Technology. Fermentation.

[65] Vilela-Moura A., Schuller D., Mendes-Faia A., Côrte-Real M. (2008). Reduction of volatile acidity of wines by selected yeast strains. Appl. Microbiol. Biotechnol..

[66] Comitini F., Gobbi M., Domizio P., Romani C., Lencioni L., Mannazzu I., Ciani M. (2011). Selected non-*Saccharomyces* wine yeasts in controlled multistarter fermentations with *Saccharomyces cerevisiae*. Food Microbiol..

[67] Ciani C., Ferraro F. (1998). Combined use of immobilized *Candida stellata* cells and *Saccharomyces cerevisiae* to improve the quality of wines. J. Appl. Microbiol..

[68] Arevalo-Villena M., Bartowsky E., Capone D., Sefton M. (2010). Production of indole by wine-associated microorganisms under oenological conditions. Food Microbiol..

[69] Magyar I., Tóth T. (2011). Comparative evaluation of some oenological properties in wine strains of *Candida stellata, Candida zemplinina, Saccharomyces uvarum* and *Saccharomyces cerevisiae*. Food Microbiol..

[70] Ciani M., Ferraro L., Fatichenti F. (2000). Influence of glycerol production on the aerobic and anaerobic growth of the wine yeast *Candida stellata*. Enzym. Microb. Technol..

[71] Van Dijken J.P., Scheffers W.A. (1986). Redox balances in the metabolism of sugars by yeasts. FEMS Microbiol. Rev..

[72] Petruzzi L., Capozzi V., Berbegal C., Corbo M.R., Bevilacqua A., Spano G., Sinigaglia M. (2017). Microbial Resources and Enological Significance: Opportunities and Benefits. Front. Microbiol..

[73] Roudil L., Russo P., Berbegal C., Albertin W., Spano G., Capozzi V. (2020). Non-*Saccharomyces* Commercial Starter Cultures: Scientific Trends, Recent Patents and Innovation in the Wine Sector. Recent Pat. Food Nutr. Agric..

[74] Zhao H., Li Y., Liu L., Zheng M., Feng Z., Hu K., Tao Y. (2022). Effects of inoculation timing and mixed fermentation with *Pichia fermentans* on *Oenococcus oeni* viability, fermentation duration and aroma production during wine malolactic fermentation. Food Res. Int..

[75] Li N., Wang L., Yin J., Ma N., Tao Y. (2022). Adjustment of impact odorants in Hutai-8 rose wine by co-fermentation of *Pichia fermentans* and *Saccharomyces cerevisiae*. Food Res. Int..

[76] Strauss M., Jolly N., Lambrechts M., Van Rensburg P. (2001). Screening for the production of extracellular hydrolytic enzymes by non-*Saccharomyces* wine yeasts. J. Appl. Microbiol..

[77] López S., Mateo J.J., Maicas S. (2016). Screening of *Hanseniaspora* Strains for the Production of Enzymes with Potential Interest for Winemaking. Fermentation.

[78] Pérez G., Fariña L., Barquet M., Boido E., Gaggero C., Dellacassa E., Carrau F. (2011). A quick screening method to identify *β*-glucosidase activity in native wine yeast strains: Application of Esculin Glycerol Agar (EGA) medium. World J. Microbiol. Biotechnol..

[79] Medina K., Boido E., Fariña L., Gioia O., Gomez M., Barquet M., Gaggero C., Dellacassa E., Carrau F. (2013). Increased flavour diversity of Chardonnay wines by spontaneous fermentation and co-fermentation with *Hanseniaspora vineae*. Food Chem..

[80] Maturano Y.P., Rodr’iguez Assaf L.A., Toro M.E., Nally M.C., Vallejo M., Castellanos de Figueroa L.I., Combina M., Vazquez F. (2012). Multi-enzyme production by pure and mixed cultures of *Saccharomyces* and non-*Saccharomyces* yeasts during wine fermentation. Int. J. Food Microbiol..

[81] Sivieri K., Favaro-Trindade C.S., Grosso C.R.F., Grimaldi R., Lerayer A.L.F. (2014). Potential probiotic characterization and in vitro evaluation of functional properties of a strain of *Wickerhamomyces anomalus* isolated from human milk. J. Appl. Microbiol..

[82] Jara C., Laurie V.F., Mas A., Romero J., Martinez-Moreno R., Acuña-Fontecilla A., Rodriguez J., Borquez R., Luchsinger C., Lopez-Miranda J. (2016). Bioactive Compounds Produced by Yeasts: Functional and Health-Protective Benefits. Appl. Microbiol. Biotechnol..

[83] Magnusson J., Ström K., Roos S., Sjögren J., Schnürer J. (2016). Anti-inflammatory and anti-cancer activities of *peptide*- and *exopolysaccharide*-producing lactic acid bacteria. J. Dairy Sci..

[84] Vega F.E., Posada F., Peterson S.W., Gianfagna T.J., Chaves F. (2015). *Penicillium* species endophytic in coffee plants and ochratoxin A production. Mycol. Prog..

[85] Jolly N.P., Augustyn O.P.H., Pretorius I.S. (2019). Non-*Saccharomyces* yeasts in wine production: Challenges and opportunities. Curr. Opin. Biotechnol..

[86] Fernandes T., Mateus P., Couto J.A. (2021). Impact of non-*Saccharomyces* yeasts on wine chemistry and flavour: A review. Trends Food Sci. Technol..

[87] Benito S., Palomero F., Calderón F., Palmero D., Suárez-Lepe J. (2014). Selection of appropriate *Schizosaccharomyces* strains for winemaking. Food Microbiol..

[88] Moreno-Arribas M.V., Carmen Polo M. (2008). Occurrence of lactic acid bacteria and biogenic amines in biologically aged wines. Food Microbiol..

[89] Morata A., Benito S., Loira I., Palomero F., González M.C., Suárez-Lepe J.A. (2012). Formation of pyranoanthocyanins by *Schizosaccharomyces pombe* during the fermentation of red must. Int. J. Food Microbiol..

[90] Wang Y., Zhao Y.C., Fan L.L., Xia X.D., Li Y.H., Zhou J.Z. (2018). Identification and characterization of *Pichia membranifaciens* Hmp-1 isolated from spoilage blackberry wine. J. Integr. Agric..

[91] Spencer J., Ragout de Spencer A., Laluce C. (2002). Non-conventional yeasts. Appl. Microbiol. Biotechnol..

[92] Vicente J., Calderón F., Santos A., Marquina D., Benito S. (2021). High Potential of *Pichia kluyveri* and Other *Pichia* Species in Wine Technology. Int. J. Mol. Sci..

[93] Lago J.C., Tavares C., Silva J.A., Araújo F.F., Guedes de Pinho P. (2019). Sequential inoculation of *Pichia kluyveri* and *Saccharomyces cerevisiae* as a strategy for enhancing flavour complexity and improving fermentation efficiency in Cabernet Sauvignon wines. Food Chem..

[94] Sun Y.H., Zhang Q.Q., Zhi Y.F., Liu J., Gao Q., Zhang T., Wang X.Q., Zhou W. (2019). Production of 2-phenylethanol by *Pichia kluyveri* using the extract from Chinese jujube as substrate. Food Sci. Biotechnol..

[95] Ramírez M., Velázquez R. (2018). The Yeast *Torulaspora delbrueckii*: An Interesting But Difficult-To-Use Tool for Winemaking. Fermentation.

[96] Lencioni L., Romani C., Gobbi M., Comitini F., Ciani M., Domizio P. (2018). Exploring the potential of *Torulaspora delbrueckii* in mixed fermentations with two *Saccharomyces cerevisiae* strains for the production of craft beers. Food Microbiol..

[97] Belda I., Ruiz J., Esteban-Fernández A., Navascués E., Marquina D., Santos A., Moreno-Arribas M.V. (2017). Microbial contribution to wine aroma and its intended use for wine quality improvement. Molecules.

[98] Chambers P.J., Rodríguez-Consuegra I. (2020). The Impact of Yeast Strain on Wine Composition and Quality. Foods.

[99] Oliveira J.M., Semedo-Lemsaddek T., Barros D.R. (2018). Molecular detection of *Wickerhamomyces anomalus* in wine spoilage. J. Food Sci. Technol..

[100] Muñoz R., González R., Benito S., Palomero F., Morata A. (2019). The impact of *Wickerhamomyces anomalus* on wine quality and safety. Foods.

[101] Du Toit M., Engelbrecht L. (2019). Lactic Acid Bacteria and Yeasts of Wine Grapes: Sensitive and Detectable Bacteria. Fermentation.

[102] International Organisation of Vine and Wine (2018). Compendium of International Methods of Wine and Must Analysis.

[103] Guzzon R. (2019). Non-sulfur dioxide microbial inhibitors for winemaking: A review. Beverages.

[104] Cappello M.S., Bleve G., Grieco F., Dellaglio F. (2017). Use of non-*Saccharomyces* yeasts and oenological tannins in red wine vinifications: Influence on colour, astringency and sensory properties. Food Microbiol..

[105] Guillamón J.M., Sabater-Molina J.M., Jiménez-Pérez A., Guerra M.I., Heras J.M. (2003). Aroma Production by Wickerhamomyces anomalus in a Model Wine: Impact of Precursors from Sweet Cherry. J. Agric. Food Chem..

[106] Fernández M., Úbeda J., Briones A. (2000). Typing of non-*Saccharomyces* yeasts with enzymatic activities of interest in wine-making. Int. J. Food Microbiol..

[107] Lleixà J., Martín V., Portillo M.d.C., Carrau F., Beltran G., Mas A. (2016). Comparison of Fermentation and Wines Produced by Inoculation of *Hanseniaspora vineae* and *Saccharomyces cerevisiae*. Front. Microbiol..

[108] Porter T.J., Divol B., Setati M.E. (2019). *Lachancea* yeast species: Origin, biochemical characteristics and oenological significance. Food Res. Int..

[109] Zott K., Thibon C., Bely M., Lonvaud-Funel A., Dubourdieu D., Masneuf-Pomarede I. (2011). The grape must non-*Saccharomyces* microbial community: Impact on volatile thiol release. Int. J. Food Microbiol..

[110] Swiegers J.H., Bartowsky P.A., Henschke P.R., Pretorius I.S. (2005). Yeasts and their importance to wine aroma—A review. S. Afr. J. Enol. Vitic..

[111] Vaquero C., Escott C., Heras J.M., Carrau F., Morata A. (2022). Co-inoculations of *Lachancea thermotolerans* with different *Hanseniaspora* spp.: Acidification, aroma, biocompatibility, and effects of nutrients in wine. Food Res. Int..

[112] Estela-Escalante W.D., Moscosa-Santillán M., González-Ramírez J.E., Rosales-Mendoza S. (2017). Evaluation of the Potential Production of Ethanol by *Candida Zemplinina* Yeast with Regard to Beer Fermentation. J. Am. Soc. Brew. Chem..

[113] Jolly N., Augustyn O., Pretorius I. (2003). The occurrence of non-*Saccharomyces* species over three vintages in four vineyards and grape musts from four production regions of the Western Cape, South Africa. S. Afr. J. Enol. Viticult..

[114] Verduyn C. (1991). Physiology of yeasts in relation to biomass yields. Antonie Van Leeuwenhoek.

[115] Portillo M.d.C., Mas A., Cantos-Villar E. (2016). Use of non-*Saccharomyces* yeasts and oak chips for white wine fermentation. Int. J. Food Microbiol..

[116] Ciani M., Comitini F., Mannazzu I., Domizio P. (2019). Controlled mixed fermentation using non-*Saccharomyces* yeasts in winemaking. Microorganisms.

[117] Renault P., Coulon J., de Revel G., Barbe J.C., Bely M. (2019). Enhancing varietal thiolic aroma production in wine using non-conventional yeast and bacteria consortia. Front. Microbiol..

[118] Grangeteau C., Gerhards D., Rousseaux S., von Wallbrunn C., Alexandre H., Guilloux-Benatier M., Dequin S., Schmitt-Kopplin P., Alexandre G. (2021). From vineyard to wine: Impact of non-*Saccharomyces* yeasts on volatile profiles and sensory properties of single-cultivar wines. Front. Microbiol..

[119] Ciani M., Comitini F. (2018). Non-*Saccharomyces* wine yeasts have a promising role in industrial biofertilization. J. Microbiol. Biotechnol. Food Sci..

[120] Liu S.Q., Pilone G.J. (2020). Non-*Saccharomyces* Yeasts in Wine Production. Yeast in Food and Beverages.

[121] Di Maio S., Polizzotto G., Di Gangi E., Foresta G., Genna G., Verzera A., Oliva D. (2019). A contribution to the study of the fermentation kinetics of mixed culture of *Candida zemplinina* and *Saccharomyces cerevisiae* in winemaking. LWT.

